# Effects of a family-focused dyadic psychoeducational intervention for stroke survivors and their family caregivers: a pilot study

**DOI:** 10.1186/s12912-022-01145-0

**Published:** 2022-12-21

**Authors:** Huanyu Mou, Stanley Kam Ki Lam, Wai Tong Chien

**Affiliations:** The Nethersole School of Nursing, Faculty of Medicine, The Chinese University of Hong Kong, Esther Lee Building, Shatin, New Territories, Hong Kong SAR, China

**Keywords:** Stroke, Family caregiver, Psychoeducation, Dyadic intervention, Functioning, Caregiver burden

## Abstract

**Background:**

Stroke is one of the leading causes of disability in China and worldwide, affecting the health and well-being of both stroke survivors and their family caregivers (i.e. stroke dyads). Dyadic interventions targeting both as active participants can be beneficial for the dyads’ health and well-being. Psychoeducation is a potentially acceptable approach to developing participants’ knowledge about their disease management to promote their recovery. This study aims to explore the feasibility, acceptability, and preliminary effects of a family-focused dyadic psychoeducational intervention for stroke dyads.

**Methods:**

This study was a single-blinded, parallel-group randomised controlled trial. Totally, a convenience sample of 40 stroke dyads was recruited. The intervention included three in-hospital face-to-face education sessions and four weekly post-discharge follow-up telephone counselling sessions. Feasibility was assessed by the rates of recruitment, attritions, and adherence to the intervention. Acceptability was evaluated via semi-structured qualitative interviews. Preliminary intervention effects were evaluated on primary (survivors’ functioning and caregivers’ burden) and secondary (caregivers’ competence and dyads’ coping, depressive and anxiety symptoms, family functioning, and dyadic relationship) outcomes.

**Results:**

Intervention feasibility was established with satisfactory recruitment (76.9%), attrition (10%), and intervention completion (75%) rates. Qualitative interviews suggested that the intervention was acceptable and useful to stroke dyads. The intervention effects on survivors’ functioning were significant in the memory and thinking (F = 8.39, *p* = 0.022, *η* = 0.18) and mobility (F = 5.37, *p* = 0.026, *η* = 0.12) domains, but not significant on their overall functioning (F = 2.39, *p* = 0.131). Caregiver burden in the intervention group was significantly greater reduced at post-test than the control group, with a large effect size (F = 7.55, *p* = 0.013, *η* = 0.28). For secondary outcomes, this intervention suggested a significant effect on caregivers’ competence (F = 5.20, *p* = 0.034, *η* = 0.22), but non-significant effects on other outcomes.

**Conclusions:**

The family-focused dyadic psychoeducation programme was feasible and acceptable for stroke dyads and showed preliminary effects for stroke dyads. These findings support a larger-scale controlled trial to further examine its intervention effects over a longer-term follow-up.

**Trial registration:**

This study was retrospectively registered as a randomised controlled trial in the ISRCTN Registry. Registration Date: October 10, 2022. Registration Number: ISRCTN18158500.

**Supplementary Information:**

The online version contains supplementary material available at 10.1186/s12912-022-01145-0.

## Background

Stroke is the leading cause of death and disability in China and worldwide [1], causing considerable physical deficits in survivors, and consequently producing a wide range of psychological distresses and social restrictions/withdrawals for both the survivors and their families [2]. Psychosocial intervention can be broadly defined as one- or multiple-component non-pharmacological intervention that focuses on psychological and/or social factors, including psychological therapies, education, training, or support [3, 4]. Different approaches to psychosocial intervention for stroke survivors and/or family caregivers have been developed and tested for improving survivors’ and/or caregivers’ psychosocial health and functional outcomes [5, 6]. Psychoeducational intervention with relevant skills training (e.g. problem-solving and self-care) appeared to be beneficial for stroke survivors’ psychological distress and family caregivers’ quality of life [5]. Several randomised controlled trials (RCTs) have also reported that psychoeducation could benefit for stroke survivors’ functional independence, caregivers’ burden and the dyads’ (survivors’ and caregivers’) family functioning for up to six months follow-up [7, 8].

Moreover, family dyadic relationship appears to be interdependent and reciprocal within a family unit [9], affecting the dyad’s health outcomes. In Chinese societies, family involvements in stroke care can be enhanced due to the norms of Confucian ideology and the recent nuclear family structure, especially attributed by the one-child policy over the past decades in mainland China [10, 11]. Family dyadic interventions targeting stroke survivor and the primary family caregiver as a unit are found to have significant improvements in not only survivors’ functionality and recovery but also in at least one caregiver outcome, such as caregiving burden [5, 6]. Therefore, interventions from the dyadic approach/perspective can be further considered to enhance the psychosocial and functional outcomes of the family dyads in stroke care.

A recent systematic review and meta-analysis of 11 RCTs investigating the effects of dyadic psychoeducational interventions for stroke survivors and their family caregivers showed inconsistent effects on most functional and psychosocial health outcomes of the dyads. This finding can be attributed or explained by the diverse characteristics of the interventions and study samples, the design of the interventions not based on a well-established theoretical framework, and not focusing on family caregivers’ caregiving needs and wellbeing [12]. The review also concluded that psychoeducational intervention initiated at the early stage of stroke recovery could exert better benefits on stroke survivors’ functional independence when being discharged home. Moreover, the combined mode of face-to-face interaction and ongoing support (e.g. telephone calls or home visits) over a follow-up period provided by health professionals (e.g. nurses and social workers) appeared to be beneficial for extending and reinforcing the intervention effects from hospitalisation to subsequent home care [5, 12]. Therefore, our research team developed a family-focused dyadic psychoeducational intervention (FDPEI) programme for stroke survivor-family caregiver dyads. This pilot study aimed to test the feasibility, acceptability and preliminary effects of the FDPEI programme on stroke dyads’ functional and psychosocial health outcomes.

## Methods

### Study design and setting

This study was a single-blinded randomised controlled trial with repeated-measures, parallel-group design conducted between December 2020 and February 2021 in one university-affiliated general hospital and one rehabilitation hospital in Jinan. Jinan is in the northeast and is the capital city of Shandong province in China with about 7 million population.

This study was retrospectively registered as a randomised controlled trial in the ISRCTN Registry (registration No.: ISRCTN18158500; first registration date: 10/10/2022).

### Participant recruitment and randomisation

A convenience sample of 40 dyads (20 per arm) was recruited according to the general rule of thumb for pilot study (≥ 30) [13], and an estimated attrition rate of 25% in studies of dyadic psychoeducational programmes in stroke care [12]. Survivors were included if they were: a) first-time diagnosed with stroke within one month; b) aged 18 years or above and willing to participate; and c) receiving daily care by a family member. Family caregivers were the unpaid family members who primarily cared and supported their stroke survivors in daily life [14]. Inclusion criteria of family caregivers were: a) one of the family members who was the primary caregiver of the survivors in families; and b) aged 18 years or above and able to give informed consent for study participation. Exclusion criteria for the study were: a) survivors having < 6-month life expectancy and high independence in activities of daily living (ADLs) with < 3 score of the modified Rankin Scale [15]; b) having comorbidities of other severe medical and/or mental disease(s); c) having visual, auditory and/or cognitive impairments (Mini-Mental State Examination, MMSE < 20 [16]), causing difficulties in following study instructions; and/or d) engaging in another psychosocial intervention research.

The potential participants (stroke dyads) were confirmed by screening their medical records and face-to-face interview for assessing their levels of dependence in ADLs, cognitive function (MMSE) and stroke and caregiving related information based on the selection criteria, and then sought for written consent for study participation. Following the baseline assessment, the participants were randomly allocated into either the intervention or the control group by drawing a labelled card in an opaque and sealed envelope from an independent research assistant not involved in the study recruitment and implementation. The labelled cards were numbered using a list of computer-generated random numbers (https://www.sealedenvelope.com/simple-randomiser/v1/lists) in blocks (size of four) to ensure balanced group memberships over time [17].

### Intervention

According to the recommendations from the Medical Research Council [18], the design of healthcare intervention with multiple interacting components can be derived from the existing evidence and the theoretical models relevant to the topic. Therefore, the FDPEI programme was developed based on the evidence from our recent systematic review on the effects of dyadic psychoeducational intervention for stroke dyads, and the theoretical underpinnings from the Double ABC-X model [12, 19]. Based on the findings from our systematic review [12], three components were included in the FDPEI programme: a) information provision, which increased the family dyads’ knowledge and helped reframe their perceptions towards stroke and its consequences; b) psychological support (e.g. counselling), which guided and facilitated the dyads towards positive emotional responses to daily stress; and c) behavioural and emotional regulation, which modified the dyads’ behaviours to promote their health outcomes.

#### Theoretical framework

**Fig. 1:**
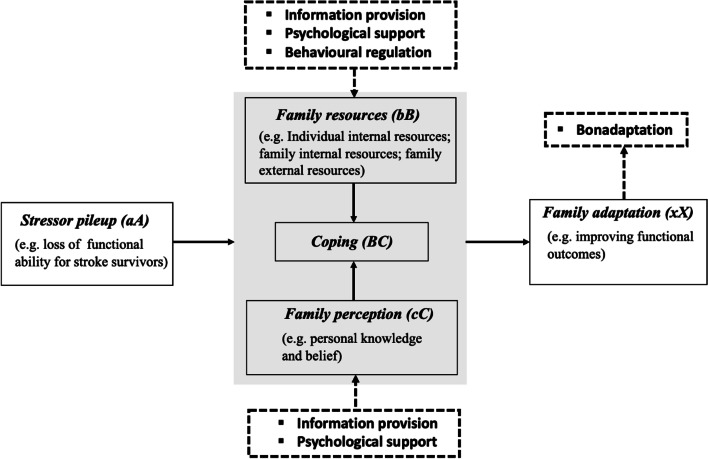
Theoretical framework of the family-focused dyadic psychoeducational intervention programme

As shown in Fig. 1, five key elements in the Double ABC-X model were included for designing the FDPEI programme, including stressor pile-up, family resources, family perception, coping, and family adaptation [19]. Based on the Double ABC-X model, the sudden occurrence of stroke can act as an initial life stressor in the family. In order to address the demands caused by stroke and its consequences, the family can adopt appropriate coping strategies based on the resources available to the family and their perceptions towards the family situations, and eventually, to achieve adaptive changes of their outcomes [20]. The designed FDPEI programme targeted at reinforcing the family resources and modifying the family dyad’s perceptions towards post-stroke hardships to enhance their coping abilities for addressing post-stroke life disturbances, thereby improving stroke dyad’s functional and psychosocial outcomes. The family resources were enhanced via providing the individual’s internal (e.g. providing information on self-care/care) and the family’s internal (e.g. behavioural regulation for improving dyads’ relationship) and external supports (e.g. providing information on community services). The family perceptions were reframed via enhancing the knowledge about stroke and care, and providing psychological support to guide them towards positive emotional responses to daily stress.

#### Delivery

This programme included two parts: Part I (three structured face-to-face education) and Part II (four weekly follow-up telephone counselling), and its protocol is presented in Table 1. Part I took place in the hospital or inpatient rehabilitation units, aiming to equip dyads with knowledge and skills relevant to stroke recovery care and help them prepare for the transition from hospital to home. It included three sessions: a) getting to know stroke, b) adaptation for care or self-care in activities of daily life, and c) psychosocial adjustment and stress management. The first session was conducted within one week after recruitment when patients’ conditions became stable. The other two sessions were run in 2–3 days intervals near discharge.

**Table 1:** Family-focused dyadic psychoeducational intervention (FDPEI) programme

Session	Goal	Content / Activities	Components/strategies
**Part I** (Structured face-to-face education)			
Session 1	To provide the overview of stroke for stroke dyads in order to lay the knowledge foundation	***Getting to know stroke*** *Content* 1) Providing dyads with information about stroke, including its aetiology, classifications, signs and symptoms, risk factors, recovery process, and post-stroke health management 2) Informing dyads about how to prevent recurrence of stroke and keep healthy lifestyle in the future 3) Encouraging dyads to express their ideas and views about stroke recovery and post-stroke life, and modifying their inappropriate belief and perceptions *Delivery mode:* Face to face/dyad *Duration:* 1 h *Format:* 45-min individualised teaching activity + 15-min discussion activity	1) Dyad’s resources and perception (information provision)
Session 2	To foster dyads’ coping ability for living in present daily life and normalising the situation in activities by providing information and skill training	***Adaptation for care or selfcare in activities of daily life*** 1) Providing dyads with information about stroke rehabilitation techniques for recovery, care/selfcare techniques in terms of their basic activities (e.g., dressing, personal hygiene, and mobility) and post-stroke complications, and the relevant coping strategies (effectively communication, actively listening, and goal setting) to help dyads implement care/selfcare and rehabilitation activities smoothly 2) Demonstration and return demonstration of the learnt care/selfcare and rehabilitation skills 3) Encouraging dyads to express or share their views or difficulties on care/selfcare and stroke rehabilitation activities, clarifying their relevant misunderstanding, and discussing the potential coping strategies to address these difficulties *Delivery mode:* Face to face/dyad *Duration:* 1 h *Format:* 30-min individualised teaching activity + 30-min skill-training activity	1) Dyads’ perception (information provision) 2) Dyads’ resources (information provision, behavioural regulation)
Session 3	To introduce the information about psychosocial disturbances after stroke and introduce some coping strategies for dyads to adapt these disturbances	***Psychosocial adjustment and stress management*** 1) Providing dyads with information about the psychosocial disturbances that they probably encountered in post-stroke lives, e.g. depression, frustration or uncertainty about future 2) Introducing some possible coping strategies to address these negative emotions (e.g. deep breathing and progressive muscle relaxation) and enhance dyads’ reciprocal relationship (e.g. expression of concern and appreciation) 3) Demonstration and return demonstration of the learnt coping techniques 4) Encouraging dyads to express and share their experience and views about psychosocial disturbances, providing psychological support, and discussing the potential strategies that they can adopt to cope with *Delivery mode:* Face to face/dyad *Duration:* 1 h *Format:* 30-min individualised teaching activity + 15-min technique-training activity + 15-min discussion activity	1) Dyads’ perception (information provision, psychological support) 2) Dyads’ resources (information provision, emotional and behavioural regulation)
**Part II** (Follow-up telephone counselling)			
	To provide stroke dyads with continuing support in their post-discharge daily lives and reinforce the in-hospital intervention effects	1) Helping dyads identify the stressful issues or psychosocial disturbances that they experienced in the past one week 2) Discussing with stroke dyads about the influence of the perceived issues in their daily lives, and discussing the potential methods to cope with the identified issues 3) Helping dyads identify the appropriate action plan, and encouraging them to implement it in the following week 4) Providing information of professional knowledge and community services links when necessary *Delivery mode:* Telephone/dyad *Duration:* 4 weeks *Frequency and sessions*: 4 contacts, with 30 min/contact	1) Dyads’ perception (information provision, psychological support) 2) Dyads’ resources (information provision, emotional and behavioural regulation)

Part II provided four weekly post-discharge telephone counselling sessions (starting from one week post-discharge) to help the dyads identify and cope with the issues encountered in their daily lives and reinforce the knowledge and skills learned in Part I. During each contact, the dyads were both encouraged to express their difficulties regarding stroke recovery and care over the past week. Their main difficulties/challenges were fully discussed with the intervener; and the best alternatives to resolving the problems were identified and encouraged to be implemented.

#### Development of information booklet

An information booklet was designed and distributed to the participants at the start of intervention as the reference material. The contents of the information booklet were consistent with those included in the three sessions of Part I. Before implementing the intervention, three stroke care specialists (one physician in stroke and two nurse specialists in stroke care) and three stroke dyads were invited to conduct an expert review of the booklet contents. All of them were asked to independently assess the clarity, relevance and adequacy of the booklet using a four-point rating scale (1 = strongly disagree, 2 = disagree, 3 = agree, and 4 = strongly agree), and provide written comments for the inappropriate/irrelevant/inadequate items (rated 1 or 2). Based on their comments, further revisions were made, including adding the practical instructions on stroke rehabilitation techniques, until reaching full agreement. Eventually, they considered the information booklet as easy-to-understand and relevant to stroke recovery and care.

#### Intervener and training

All intervention sessions/calls were delivered by a registered nurse with backgrounds in psychiatric nursing, general medical nursing and chronic disease care. To better understand the stroke family care and rehabilitation services in the current local system, the intervener had learned the knowledge and skills relevant to the intervention, and had extensive discussions with stroke care experts and clinicians before implementing the intervention.

### Control group

All stroke dyads were provided with routine stroke care in the general or rehabilitation hospitals under study, including medical treatments from doctors, rehabilitation care from physiotherapists or occupational therapists, and general care and health education from general nurses.

### Data collection and outcome measurements

Study data were collected at baseline (T_0_) and immediately post-intervention (i.e. four weeks post-discharge) (T_1_). Before randomisation, the first researcher collected stroke dyads’ sociodemographic and clinical characteristics, and baseline outcome assessments via extracting data from medical records and a face-to-face interview. At T_1_, data were collected via telephone by two research assistants trained by the researchers and blinded to the randomisation and group assignment (during the COVID-19 pandemic and considering the participants’ time/venue convenience). Inter-rater reliabilities of outcome measures between the research assistants were assessed with ten family dyads and found acceptable before use (Intra-class correlation coefficients: 0.68–0.94) [21].

#### Feasibility

Intervention feasibility was determined by examining participant recruitment, attrition and intervention adherence rates. Intervention completions were assessed in terms of completing > 60% of the programme (2–3 sessions in Part I and 3–4 telephone calls in Part II) [22]. An activity log sheet (Appendix A) was used for the intervener to record the important events, questions and difficulties encountered during each session/call. An intervention fidelity checklist (Appendix B) was completed by the intervener by the end of each session/call to monitor the intervention delivery and performance. The ratings of the checklist items were discussed among the researchers to identify the strengths and weaknesses, and areas for improvements during the coming sessions and future use.

#### Acceptability

Acceptability can be regarded as a multi-facet construct that assesses the extent to which people receiving (or delivering) intervention perceive the intervention to be appropriate based on their anticipated or experienced responses to the intervention [23]. In this study, the acceptability assessment at immediately post-intervention mainly focused on participants’ experience from intervention initiation to completion, including their attitudes, perceived benefits, and difficulties or burdens encountered in the intervention participation. The acceptability of participants was evaluated via individual semi-structured interviews for participants in the intervention group using several open-ended questions (Appendix C). Each pair of stroke survivors and family caregivers in the intervention group were interviewed separately in individual basis within one week post-intervention by another trained research assistant with rich experience in qualitative interviews; and the interview data were digitally recorded.

#### Outcome measures

The selection of outcome measures was based on the constructs of the adopted theoretical framework and outcome measures identified from our recent systematic review [12, 19]. The primary outcomes included survivors’ functioning and caregiver burden. The secondary outcomes included caregivers’ caregiving competence, and dyads’ coping, depressive symptoms, anxiety symptoms, family functioning and dyadic relationship.

*Survivors’ functioning*. Stroke Impact Scale (SIS) Version 3 was used to comprehensively evaluate patients’ perceived functioning impact by stroke, which contained eight domains (i.e. strength, memory/thinking, emotion, communication, ADL, mobility, hand function and social participation) [24]. This scale consists of 59 items on a scale of 1 to 5. Aggregated scores of total score and domains ranged from 0 to 100, with a higher score indicating better functioning perceived by stroke survivors. The translated Chinese-version SIS had good internal consistency (Cronbach’s alpha = 0.92) and test–retest reliability coefficient (0.90) [25].

*Caregiver burden*. Caregiver Burden Inventory was adopted to measure the impact of burden on caregivers [26]. This scale contains 24 items in the five domains of time dependence, development, physical health, emotional health, and social relationship. Each item was rated using a five-point Likert scale. The total score ranges from 0 to 96, with a higher score indicating more burden. The Chinese-version CBI was validated and suggested a satisfactory reliability with Cronbach’s alpha coefficient of 0.85 [27].

*Coping*. The Family Crisis-Oriented Personal Evaluation Scale (F-COPES) was used to measure the family problem-solving and coping behaviours in response to the stressful situations [28]. It contains five domains, namely, acquiring social support, reframing, seeking spiritual support, mobilising family to acquire and accept support, and passive appraisal. The F-COPES included 29 items on a five-point Likert scale, with higher scores indicating greater problem-solving and coping ability. It has been shown to have good validity and reliability with a Cronbach’s alpha of 0.86 in family caregivers of patients with dementia in China [29].

*Caregiving competence*. Caregiving Competence Scale (CCS) is a four-item scale to evaluate the adequacy of participants’ performance as caregivers [30]. Each item is scored on a scale of 1 to 4, with higher score indicating more competent caregiving. This scale was translated into Chinese and validated by Cheng and colleagues [31], which showed good internal consistency (Cronbach’s alpha: 0.81).

*Depressive symptoms.* Depressive symptoms were evaluated using the Patient Health Questionnaire-9 (PHQ-9), containing nine items on a scale of 0 to 3 [32]. The total score ranges from 0 to 27, with a higher score indicating more severe depressive symptoms. The PHQ-9 has been well validated in Chinese and showed good reliability with a Cronbach’s alpha of 0.86 [33].

*Anxiety symptoms*. Generalised Anxiety Disorder-7 (GAD-7) was adopted to evaluate the anxiety symptoms, including seven items on a 4-point Likert scale (from 0 to 3) [34]. Higher scores indicated more severe anxiety symptoms. The translated Chinese-version GAD-7 showed good validity and reliability with a Cronbach’s alpha coefficient of 0.90 in hospital outpatients [35].

*Family functioning*. The family functioning was measured via the general functioning subscale of Family Assessment Device (GF-FAD) [36], with 6 items evaluating healthy family functioning and 6 items evaluating unhealthy family functioning. Each item is on a 4-point Likert scale. The total score (from 1 to 4) was obtained via averaging the 12 responses, with lower score suggesting better family functioning. The Chinese GF-FAD has been validated and showed satisfactory internal consistency (Cronbach’s alpha coefficient = 0.70) in Chinese college students [37, 38].

*Dyadic relationship*. The Mutuality Scale (MS) was used to measure the perceived relationship of emotional investment and mutual support between dyads [39], including the four domains of love, shared pleasurable activities, shared value, and reciprocity. The MS consists of 15 items on a scale of 0–4. The total score is generated by averaging the 15 responses, ranging from 0 to 4. Higher scores indicate better relationship quality between dyads. The MS has been suggested to be valid and reliable in people with stroke [9, 40]. The Chinese MS was also well validated and showed good internal consistency (Cronbach’s alpha = 0.94) [41].

### Data analysis

All quantitative data were analysed using IBM SPSS 23.0, with the significance level at 0.05 (two-tailed). Descriptive statistics were used for summarising all socio-demographic and clinical characteristics, the study process and outcome scores. Intention-to-treat principle was adopted for analysing outcome data by using the last observation carried forward method to handle missing data [42]. All continuous data were checked for normal or nearly normal distributions according to the Q-Q plots and values of skewness and kurtosis. Therefore, homogeneity of the participants’ characteristics and outcome scores between groups at baseline (T_0_) was examined by independent *t* tests and Chi-square/Fisher’s exact tests. The interaction (group × time) effects on individual outcomes were assessed using the two-way ANOVA. Partial eta squared (*η*) was used to interpret effect sizes, including small (*η* ≥ 0.01), median (*η* ≥ 0.06) and large (*η* ≥ 0.14) effect [43].

The qualitative data were analysed using the content analysis [44], which was conducted by the first author and another research assistant who were bilingual (English and Mandarin) and had experience in qualitative data analysis. All digitally audio-recorded interview data were transcribed verbatim by the first author and checked by the research assistant. First, they independently read the transcribed materials repeatedly, and generated the codes manually using colouring pens. Then, the different coloured codes were compared for similarities and differences, and sorted into tentative themes and subthemes. After that, the themes and subthemes were reached consensus via discussion between them. Eventually, all themes, subthemes and examples were translated into English by the first author, and the semantic equivalence was cross-checked by the research assistant.

## Results

### Feasibility

**Fig. 2:**
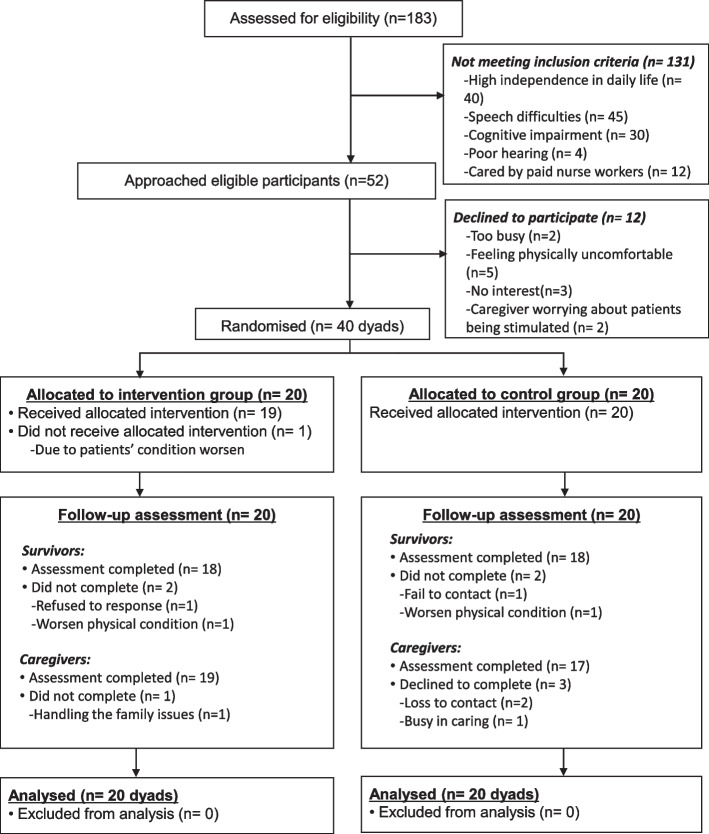
The CONSORT flow chart of the pilot study

As shown in Fig. 2, among 52 dyads who were eligible for recruitment, 40 dyads agreed to participate (recruitment rate = 76.9%). The attrition rates were 10% given that four survivors and four caregivers (two family dyads, and two survivors and two caregivers from different families) did not complete the post-test. In addition, 15 of 20 dyads (75%) completed the FDPEI programme; of whom, five dyads fully attended three sessions in Part I and four contacts in Part II. The main incompletion reasons were early discharge from hospitals (*n* = 6) or unanswered post-discharge telephone calls (*n* = 4). According to the activity log, the intervention average attendance rate was 75% (mean = 2.25 sessions) in Part I, and 78.8% (mean = 3.15 contacts) in Part II. The programme was implemented smoothly, and no great difficulties or adverse events occurred during the study. According to the intervention fidelity checklist self-assessed by the intervener, the average adherence to the intervention protocol or its items (for four FDPEI subgroups) were 100%, 94.7% and 93.8% for the three sessions in Part I accordingly, and 100% for the counselling sessions in Part II.

### Acceptability

Among the 20 dyads in the intervention group, 10 stroke survivors (mean age = 50.0 years, 3/10 females), and 11 caregivers (mean age = 44.5 years, 7/11 females, and 9/11 spouses) agreed and completed the interviews. The reasons for those not involved in the interviews included: failing to contact (5 survivors, 5 caregivers), being occupied by family issues (4 stroke dyads), and difficulty in solely communicating by telephone (1 survivor). The main themes, sub-themes and supporting verbatim examples were summarised in Table 2.

**Table 2:** Perceptions of stroke dyads for participating in the FDPEI programme

	**Stroke survivors**	**Family caregivers**
***Positive attitudes towards the intervention***		
Helpful for stroke recovery and care	S3: ‘It’s definitely helpful. Especially, I read the booklet from time to time.’ S6: ‘What I learn from you is indeed what I need after I have a stroke.’	C2: ‘Of course, I think it is helpful for his recovery…sometimes, we read the information booklet as a reference for his recovery.’ C5: ‘Even though I cannot comprehensively tell you the details of what I’ve learned from the intervention at this moment, I can recall something relevant when I encounter a specific issue in my daily life.’
Practical for improving post-stroke daily life	S6: ‘I use those relaxation techniques a lot. You know, I had a bad temper. Now, I know I need to control my emotions. So when I encounter something unpleasant, I use these techniques to relax and adjust myself, like deep breathing.’	C10: ‘To be honest, I’m not interested in the written materials with many words, so did my grandma [stroke survivors]. I think … I just want to know how I can do straightforwardly … now, it [information booklet] is good, since it contains many pictures for demonstration.’ C5: ‘… I prefer to interact with you. I can ask some issues I encountered and discuss with you. And then, I can know more about what we need. I think it’s much better than words on the ward [health education posters in the ward]. You know, each patient has different symptoms, so they have their own specific needs.’
***Perceived benefits***		
Increased knowledge on stroke recovery and care	S1: ‘Now, I realise that I cannot be completely cured at once, and the rehabilitation is a slow process … as said in the hospital slogan “healing” in life, living in healing (*在生活中康復, 在康復中生活*)”.’ S9: ‘I think I know more knowledge about what I can do for recovery and prevention of stroke in the future.’	C5: ‘After he was diagnosed with stroke, I search many information online. But lots of them were neither comprehensive nor reliable … I think you are a professional. I feel that I’ve mastered many key points on how to care for him [survivor], like modifying unhealthy lifestyles, regulating our stress and emotions.’
Enhanced coping ability for recovery/caring	S6: ‘Sometimes, I’m lazy to exercise my impaired body at home … after we discussed last time, I set a small goal for myself and complete it every day. I think it’s effective.’	C11: ‘Sometimes, I check the information booklet when I encounter a problem, and then, I probably know how to cope with it.’
Emotional support	S6: ‘At our age [60 +], the opportunities to contact others, even relatives or friends, become less and less … you are just a stranger; you call me often and are concerned for me. I feel so happy.’	C8: ‘It warms my heart that you care for us even after we left the hospital.’
Improved communication and interpersonal relationship	S9: ‘I try to communicate with her [family caregiver] and tell her more about my thoughts … I know she is also stressed. I try to be cheerful in case she worries about me.’	C6: ‘At first, I’m struggling on how to help him recover and what I can do for him … I’ve changed my mind now. I prefer to discuss with him and involve him in the decision of his own life.’
More focus on self-care (for caregivers just)		C6: ‘I have to focus on my own health as well … I try to modify my own lifestyle, such as doing some exercise, reading … I have to become stronger so that our home will not fall apart.’
***Suggestions for improvements***		
Longer time intervals between education sessions	S6: ‘For the education frequency, the present frequency is ok for me. But I think twice a week might be more acceptable.’	C7: ‘I think the frequency of education in hospital is high, so I felt a little burdened. Maybe once a week is greater.’
More home-based rehabilitation strategies	S9: ‘I think more details about rehabilitation strategies could be included in the intervention, especially about those we can do at home.’	C6: ‘For now, most of us are still focused on his physical recovery. Yes, the psychological adjustment is also important, but since he has a chance to recover better, I hope to know more specific and effective strategies to help him in rehabilitation at home.’

#### Positive attitudes towards the intervention

Overall, most of the participants (dyads) showed positive attitudes towards the participation in FDPEI. First, most survivors and caregivers said that the intervention was helpful for them in the process of stroke recovery and care. For example, some of them reported that questions encountered at home could be solved promptly via discussing with the intervener. Moreover, they indicated that the intervention was practical for adoption in daily life. Especially, the format of face-to-face interaction education mode was more appropriate than written materials, as they could know more based on their own needs.

#### Perceived benefits

Several benefits of the FDPEI programme were found via the interviews. The benefits perceived by both survivors and caregivers included increasing knowledge on stroke recovery and care, enhancing coping abilities for challenges/difficulties in stroke selfcare and recovery, providing emotional support, and improving communication and relationships with their counterparts. Besides, some family caregivers also mentioned that they were willing to pay more attention on their own health and selfcare after the study.

#### Suggestions for improvements

In terms of the difficulties and suggestions for completing the intervention, several participants suggested to reschedule the education sessions with longer time intervals (e.g. once or twice a week), as they thought the current education frequency (two or three days between education sessions) was a little burdened. Besides, some of them recommended to improve this programme by introducing more home-based rehabilitation strategies based on their own conditions. Apart from these comments, no main difficulties in the intervention and study participation were reported by the participants.

### Preliminary effects

There were no significant between-group differences in participants’ sociodemographic and clinical characteristics, and outcome scores at T_0_ (*p* = 0. 065–1.000) (Tables 3 and 4). The results of two-way ANOVA tests (Table 4) indicated that the interaction (group × time) treatment effects were significant in the domains of memory/thinking (F = 8.39, *p* = 0.022) and mobility (F = 5.37, *p* = 0.026) of the survivors’ functioning with large and medium effect sizes (*η* = 0.18 and 0.12, respectively), despite having non-significant result in their overall functioning (F = 2.39, *p* = 0.131). The FDPEI group indicated a significantly greater reduction in caregiving burden than the control group with a large effect size (F = 7.55, *p* = 0.013, *η* = 0.28). In addition, the FDPEI participants reported a significantly greater increase in caregiving competence, compared with the control group (F = 5.20, *p* = 0.034, large effect size, *η* = 0.22). However, there were no significant between-group differences in dyads’ coping, depressive and anxiety symptoms, family functioning, and dyadic relationship (*p* = 0.085–0.814).

**Table 3:** The comparisons of participants’ sociodemographic and clinical characteristics at T_0_

*Stroke survivors*					*Family caregivers*				
	FDPEI	Control	χ^2^/t	*p*		FDPEI	Control	χ^2^/t	*p*
Gender			0.00	1.000	Gender			0.10	1.000
Male	14(70%)	14(70%)			Male	11 (55%)	10 (50%)		
Female	6(30%)	6(30%)			Female	9 (45%)	10 (50%)		
Age	49.95 ± 11.51	53.85 ± 11.28	1.08	0.286	Age	45.61 ± 12.14	48.10 ± 12.20	0.63	0.533
Marital status			0.00^#^	1.000	Marital status			0.36	1.000
Married	19(95%)	19(95%)			Married	19 (90%)	18 (90%)		
Single/widowed	1(5%)	1(5%)			Single/widowed	1 (5%)	2 (10%)		
Education level			0.40	0.752	Education level			0.00	1.000
Primary school or below	9 (45%)	11 (55%)			Primary school or below	10 (50%)	10 (50%)		
Secondary school and above	11 (55%)	9 (45%)			Secondary school or above	10 (50%)	10 (50%)		
Employment			1.67	0.333	Employment			2.13	0.273
Employed	14 (70%)	10 (50%)			Employed	17(85%)	13(65%)		
Unemployed/retired	6 (30%)	10 (50%)			Unemployed/retired	3(15%)	7(35%)		
Financial condition			2.71^#^	0.304	Relationship with survivors			1.72^#^	0.480
Good	4 (20%)	7 (35%)			Spouse	13(65%)	9 (45%)		
Fair	15 (75%)	10 (50%)			Parent/Children	6 (30%)	9 (45%)		
Poor	1 (5%)	3 (15%)			Others	1(5.0%)	2(10%)		
Self-rated health			4.33	0.119	Living with survivors			0.00	1.000
Good	5 (25%)	6 (30%)			Yes	15(75%)	15(75%)		
Fair	10 (50%)	4 (20%)			No	5(25%)	5(25%)		
Poor	5 (25%)	10 (50%)			Location			0.11	1.000
Stroke type			1.14^#^	0.695	Village	8 (40%)	7 (35%)		
Ischemia	15 (75%)	13 (65%)			Urban	12 (60%)	13 (65%)		
Haemorrhage	4 (20%)	4 (20%)			Finance			0.32^#^	1.000
Unspecific	1 (5%)	3 (15%)			Good	3 (15%)	3 (15%)		
Hemiplegia			2.89^#^	0.307	Fair	13 (65%)	14 (70%)		
Left	12 (60%)	10 (50%)			Poor	4 (20%)	3 (15%)		
Right	8 (40%)	7 (35%)			Self-reported health			1.18^#^	0.752
Both	0 (0%)	3 (15%)			Good	8 (40%)	11 (55%)		
Hypertension			0.14^#^	1.000	Fair	11 (55%)	8 (40%)		
Yes	16 (80%)	15 (75%)			Poor	1 (5%)	1 (5%)		
No	4 (20%)	5 (25%)			Previous care experience			1.11^#^	0.605
Diabetes Mellitus			0.63^#^	0.695	Yes	1 (5%)	3 (15.0%)		
Yes	3 (15%)	5 (25%)			No	19 (95%)	17 (85.0%)		
No	17 (85%)	15 (75%)			Co-carer			0.24	0.751
Physical dependence			0.58	0.910	Yes	10 (50%)	11 (57.9%)		
Moderate	4 (20%)	6 (30%)			No	10 (50%)	8 (42.1%)		
Moderate severe	12 (60%)	10 (50%)							
Severe	4 (20%)	4 (20%)							
Stroke onset days			4.80	0.065					
≤ 2 weeks	18 (90%)	12 (60%)							
> 2 weeks to 1 month	2 (10%)	8 (40%)							

^#^: Fisher’s exact test

**Table 4:** Study outcome scores of stroke survivors and family caregivers

	Group	FDPEI (*n* = 20)	Control (*n* = 20)	Comparison at T_0_		Two-way ANOVA						
		Mean (SD)	Mean (SD)	t	*p*	Group effect		Time effect		Interaction effect		
						F	*p*	F	*p*	F	*p*	*η*_*p*_^*2*^
***Stroke survivors***												
SIS	T_0_	42.82 (8.81)	44.87 (11.55)	0.63	0.531	0.12	0.730	22.43	< 0.001	2.39	0.131	0.06
	T_1_	57.20 (18.74)	52.18 (19.44)									
Strength	T_0_	22.50 (16.77)	24.38 (19.54)	0.33	0.747	0.02	0.878	18.39	< 0.001	0.10	0.754	0.00
	T_1_	38.44 (21.49)	38.13 (19.54)									
Memory and thinking	T_0_	72.50 (18.29)	73.04 (19.30)	0.09	0.929	1.83	0.219	3.85	0.193	8.39	0.022	0.18
	T_1_	84.29 (18.52)	69.64 (24.97)									
Emotion	T_0_	61.53 (12.53)	54.86 (17.95)	-1.36	0.181	0.69	0.412	1.01	0.322	0.73	0.398	0.02
	T_1_	62.08 (22.50)	61.81 (16.59)									
Communication	T_0_	92.32 (12.60)	90.36 (11.77)	-0.51	0.613	2.04	0.161	3.11	0.086	1.99	0.166	0.05
	T_1_	91.43 (16.93)	82.32 (16.53)									
Activities of daily living	T_0_	31.63 (8.59)	32.88 (14.08)	0.34	0.737	0.02	0.886	30.15	< 0.001	0.02	0.882	0.00
	T_1_	50.50 (23.93)	50.75 (26.01)									
Mobility	T_0_	18.06 (19.06)	28.75 (23.71)	1.57	0.124	0.01	0.907	29.96	< 0.001	5.37	0.026	0.12
	T_1_	50.97 (33.63)	42.08 (31.92)									
Hand function	T_0_	6.25 (11.34)	12.25 (15.00)	1.43	0.162	0.27	0.610	18.83	< 0.001	0.32	0.575	0.01
	T_1_	29.00 (34.44)	29.75 (32.30)									
Social participation	T_0_	27.34 (11.91)	32.97 (19.03)	1.12	0.270	0.01	0.935	3.18	0.083	2.39	0.131	0.06
	T_1_	40.47 (26.68)	33.91 (26.68)									
F-COPES	T_0_	87.30 (7.16)	86.15 (8.13)	-0.48	0.638	0.50	0.486	46.77	< 0.001	0.38	0.540	0.01
	T_1_	98.15 (12.30)	95.20 (12.47)									
PHQ-9	T_0_	7.60 (4.15)	7.15 (5.93)	-0.28	0.782	0.03	0.865	0.84	0.364	0.56	0.460	0.01
	T_1_	6.15 (4.06)	7.00 (4.07)									
GAD-7	T_0_	5.55 (2.78)	3.80 (3.05)	-1.90	0.066	2.14	0.152	0.03	0.874	1.08	0.305	0.03
	T_1_	4.80 (3.44)	4.35 (3.07)									
GF-FAD	T_0_	2.15 (0.36)	2.11 (0.21)	-0.40	0.691	0.07	0.792	13.88	0.001	0.45	0.505	0.01
	T_1_	1.74 (0.58)	1.83 (0.43)									
MS	T_0_	2.55 (0.59)	2.63 (0.51)	0.50	0.622	0.34	0.563	0.72	0.403	1.86	0.180	0.05
	T_1_	2.82 (0.61)	2.57 (0.61)									
***Family caregivers***												
CBI	T_0_	44.30 (18.32)	42.50 (16.29)	-0.33	0.744	1.22	0.283	4.14	0.056	7.55	0.013	0.28
	T_1_	32.45 (15.86)	44.40 (16.50)									
Time dependence	T_0_	14.70 (5.08)	15.15 (3.34)	0.33	0.743	1.90	0.184	12.20	0.002	3.00	0.099	0.14
	T_1_	11.10 (6.40)	14.30 (4.52)									
Development	T_0_	12.40 (5.73)	12.15 (5.28)	-0.14	0.887	0.36	0.555	4.32	0.051	1.25	0.277	0.06
	T_1_	9.55 (4.74)	11.45 (4.80)									
Physical health	T_0_	7.95 (4.05)	6.05 (3.93)	-1.51	0.140	0.02	0.890	0.04	0.836	9.92	0.005	0.34
	T_1_	5.80 (4.10)	8.00 (4.13)									
Emotional health	T_0_	4.65 (3.91)	4.40 (3.86)	-0.20	0.840	0.21	0.649	0.15	0.704	1.04	0.320	0.05
	T_1_	3.70 (2.92)	4.75 (3.88)									
Social relationship	T_0_	4.60 (4.17)	4.75 (3.70)	0.12	0.905	5.18	0.035	0.40	0.537	4.82	0.041	0.20
	T_1_	2.30 (3.39)	5.90 (4.70)									
CCS	T_0_	10.85 (2.16)	11.70 (1.84)	1.34	0.188	0.24	0.629	0.80	0.382	5.20	0.034	0.22
	T_1_	11.75 (2.51)	11.40 (1.35)									
F-COPES	T_0_	93.55 (9.96)	91.15 (11.03)	-0.72	0.475	2.82	0.109	4.14	0.056	0.67	0.423	0.03
	T_1_	98.75 (7.65)	93.40 (8.52)									
PHQ-9	T_0_	6.10 (4.08)	6.10 (4.48)	0.00	1.000	0.03	0.870	0.12	0.734	0.06	0.814	0.00
	T_1_	5.65 (4.43)	6.00 (3.55)									
GAD-7	T_0_	5.25 (3.52)	5.70 (5.27)	0.32	0.753	0.69	0.418	3.39	0.081	0.16	0.694	0.01
	T_1_	3.45 (3.39)	4.65 (4.07)									
GF-FAD	T_0_	2.06 (0.31)	1.98 (0.33)	-0.82	0.416	0.80	0.382	5.80	0.026	3.31	0.085	0.15
	T_1_	1.66 (0.57)	1.90 (0.45)									
MS	T_0_	1.65 (0.25)	1.58 (0.26)	0.20	0.839	0.17	0.684	0.18	0.677	1.58	0.225	0.08
	T_1_	1.33 (0.46)	1.52 (0.36)									

*CBI* Caregiver burden inventory, *CCS* Caregiver competence scale, *F-COPES* Family crisis oriented personal evaluation scale, *FDPEI* Family-focused dyadic psychoeducational intervention, *GF-FAD* General functioning of family assessment device, *GAD-7* Generalised anxiety disorder scale-7, *PHQ-9* Patient health questionnaire-9, *MS* Mutuality scale, *SD* Standard deviation, *SIS* Stroke impact scale

## Discussion

The findings of this study suggest that the FDPEI was feasible and acceptable for stroke dyads, with satisfactory subject recruitment and attrition and intervention adherence rates at immediately post-intervention. This pilot study also provides preliminary evidence about the effects of the FDPEI on improving survivors’ functioning in memory/thinking and mobility and caregiver burden and benefits in caregiving competence. However, there were no significant improvements in the family dyads’ coping, depressive and anxiety symptoms, family functioning, and dyadic relationship for the FDPEI.

The recruitment rate of this study (76.9%) was the highest in comparison with other psychoeducational studies for stroke dyads (i.e. 67%-76%) [7, 45]. The attrition rate in this study (10%) was lower than previous similar psychoeducational programme in stroke care (16%-19%) [8, 45, 46]. The low drop-outs might be ascribed to that participants’ positive views and attitudes towards participating in the FDPEI, as supported by the qualitative findings (e.g. helpful for recovery and care), which facilitated their motivation for engagement. Providing accessible and early support for the family after the onset of acute or critical illness might motivate them to effectively cope with the illness and its negative consequences [47]. Furthermore, the relatively short intervention duration (< 2 months) of the FDPEI programme might be another important reason for higher rates of completing the study than those previous similar programmes (6—12 months) [8, 12, 46].

The qualitative findings elicited much positive feedbacks (e.g. helpful for stroke recovery and care) on intervention participation. The FDPEI programme was well accepted by most family dyads. Nevertheless, a few areas of improvements could be considered according to participants’ suggestions (including extending the education intervals and adding home-based rehabilitation strategies) and feasibility of intervention implementation. However, for the in-hospital education intervals, delivering three education sessions on a weekly or biweekly basis was not that feasible. Because the common hospital-stay period for acute stroke hospitalisation was usually 7–14 days in China and overseas [48], and there are sometimes special hospitalisation arrangements based on patients’ own expectations (e.g. two or three days earlier than the normal schedule). Therefore, future studies can consider flexibly adjusting the delivery schedule or giving priority to some intervention contents based on patients’ individual conditions and discharge arrangement to further enhance intervention completion sessions during the transition period.

Adding more contents and strategies of home-based stroke rehabilitation was perceived as another important aspect for improving the FDPEI programme, probably due to that patients at the early stage post-stroke usually had high expectations of achieving full recovery of the impaired functioning via optimal and effective treatments, early intervention(s) and rehabilitation plans and strategies [49]. However, in mainland China and most Asian countries, limited supporting and rehabilitative community care services could be accessed and available to these survivors and their family caregivers [50]. Therefore, future research is recommended to include more feasible and detailed rehabilitation contents (e.g. range of motion exercises) and strategies (e.g. purposive goal-directed stepping) that can be implemented at home based on the survivors’ conditions and needs. More active discussions with health professionals on family-based stroke recovery and rehabilitation at home environment can also be encouraged and facilitated in the FDPEI programme.

Regarding the preliminary effects, this study revealed a non-significant effect on the survivors’ functioning, but significant effects on the domains of their memory/thinking and mobility. These significant improvements might be attributed to the introduced knowledge related to stroke and its consequences (e.g. impaired mobility) and relevant skills training (e.g. mobility rehabilitation) via in-hospital education, as well as the strategies in helping identify and address the post-discharge issues via telephone counselling. Ostwald’s (2014) study suggested that the activities of education and interactions in a home-based psychoeducation programme might provide adequate stimulations for stroke survivors to exercise and regain their impaired capabilities of memory and thinking [46]. In line with another pilot study adopting psychoeducation and discussion about strategies in promoting physical and social activities for stroke survivors [51], remarkable improvements in the survivors’ mobility might also be due to that mobility, as a prioritised focus in post-acute stroke recovery [52], was usually preferentially targeted in post-stroke problem identification and discussion of alternative strategies over the study. Nevertheless, given that survivors’ overall functioning was a multi-faceted concept, the varied effects on different domains induced by the FDPEI might weaken the sensitivity of detecting the significant changes of overall functioning. The non-significant effects on most domains of functioning might be related to the small sample size and a variety of stroke-related characteristics (e.g. stroke severity and lesion of brain) of the included participants. Future study can further test the intervention effects via a larger-scale sample size and sample with diverse stroke-related characteristics.

For caregiver burden, the FDPEI could produce significant reductions than those receiving usual care, which was consistent with other family-based psychoeducational interventions for stroke caregivers [7, 53]. The FDPEI programme equipped caregivers with essential caregiving-related knowledge and relevant skills. Moreover, the dyadic mode encouraged caregivers to disclose and cope with their needs and concerns regarding caregiving and their own health, which consequently alleviated their perceived caregiving burden and distress. Besides, the significantly large effect on caregiving competence was also in line with the previous psychoeducational study for stroke caregivers [53], with reinforcing caregivers’ self-confidence to manage various novel situations by increasing caregiving-related knowledge and skills [54]. These significant outcomes on caregivers were also echoed by the qualitative results of this study, with emphasising that caregivers appropriately focused on their own health needs and care post-intervention.

Although the qualitative results revealed some perceived benefits induced by the FDPEI, such as improving the dyads’ coping abilities and interpersonal relationships, non-significant effects were detected on dyads’ coping, depressive and anxiety symptoms, family functioning and dyadic relationship. Eight dyads (40%) in this study missed the last in-hospital education session (psychosocial adjustment and stress management) due to early discharge arrangement, which might be a primary reason for not detecting significant between-group differences. Besides, the low levels of psychological symptoms (e.g. depressive and anxiety symptoms) at baseline possibly caused the difficulty in demonstrating statistically significant changes or improvements in their scores (floor effects). In addition, the small sample size, convenience sampling method, short follow-up period, and limitations in data analysis methods (non-covariance analysis) might also explain these non-significant intervention effects. Therefore, future full-scale RCT is recommended to adopt the aforementioned strategies (e.g. using more flexible schedule for education sessions) to further reinforce intervention attendance and completion among a large-scale sample with more diverse psychosocial characteristics and adequate study power over a reasonable follow-up period.

There are a few limitations in this study. First, the small-sized convenience sample recruited from two hospitals in one city in mainland China could reduce the study power for detecting significant intervention effects (internal validity) and limit the generalisability of study findings (external validity). In addition, adopting a self-assessed and newly developed checklist for monitoring intervention fidelity in terms of intervener’s performance might cause subjective bias and reporting bias, even though these self-report methods were cost and time saving and user-friendly [55].

## Conclusions

In sum, the findings of this pilot study support the feasibility and acceptability of the FDPEI programme for Chinese survivor-(family) caregiver dyads in stroke care. Preliminary effects/benefits were revealed on survivors’ functioning in memory/thinking and mobility, and caregivers’ burden and caregiving competence. However, no significant effects were suggested on dyads’ coping, depressive and anxiety symptoms, family functioning and dyadic relationship. The FDPEI is a potentially promising approach to enhancing stroke dyads’ functional and psychosocial health outcomes. This pilot study sampled a small size of family dyads in stroke care with few intervention incompletions and attritions. Future full-scale RCT is recommended to assess the longer-term effects of the FDPEI with a larger number of randomised family dyads in stroke care and more diverse sample characteristics, based on the refined FDPEI protocol resulted from this pilot study.

## Supplementary Information

**Additional file 1:** **Appendix A.** Activity log sheet of the FDPEI programme.

**Additional file 2:** **Appendix B.** Intervention fidelity checklist.

**Additional file 3:** **Appendix C.** The open-ended questions of qualitative interview.

## Data Availability

The datasets generated and/or analysed during the current study are not publicly available due to the need to maintain anonymity of participants and the confidentiality of the data. However, the datasets was available from the corresponding author on reasonable request.

## Abbreviations

ADLs: Activities of daily living
FDPEI: Family-focused dyadic psychoeducational intervention
MMSE: Mini-mental state examination
RCTs: Randomised controlled trials
