# Supplementary material for: Effects of a family-focused dyadic psychoeducational intervention for stroke survivors and their family caregivers: a pilot study
Source: BMC Nurs. 2022 Dec 21;21:364. doi: 10.1186/s12912-022-01145-0 (PMC9768401; doi:10.1186/s12912-022-01145-0)
Supplement: Supplementary file 1 — Additional file 1: Appendix A. Activity log sheet of the FDPEI programme. [file 12912_2022_1145_MOESM1_ESM.docx]

Appendix A. Activity log sheet of the FDPEI programme

| ID |  | Survivor name | |  | Gender |  | Age |  |
| --- | --- | --- | --- | --- | --- | --- | --- | --- |
|  |  | Caregiver name | |  |  |  |  |  |
| Survivor clinical characteristics | |  | |  | | | | |
| **Part I Session 1** | | | | - Completed □ Uncompleted | | | | |
| Date | Duration | | Attendee | Problems encountered | Other note | | | |
|  |  | |  |  |  | | | |
| **Part I Session 2** | | | | - Completed □ Uncompleted | | | | |
| **Date** | Duration | | Attendee | Problems encountered | Other note | | | |
|  |  | |  |  |  | | | |
| **Part I Session 3** | | | | - Completed □ Uncompleted | | | | |
| Date | Duration | | Attendee | Problems encountered | Other note | | | |
|  |  | |  |  |  | | | |
| **Part II Call 1** | | | | - Completed □ Uncompleted | | | | |
| Date | Duration | | Attendee | Problems encountered | Other note | | | |
|  |  | |  |  |  | | | |
| **Part II Call 2** | | | | - Completed □ Uncompleted | | | | |
| Date | Duration | | Attendee | Problems encountered | Other note | | | |
|  |  | |  |  |  | | | |
| **Part II Call 3** | | | | - Completed □ Uncompleted | | | | |
| Date | Duration | | Attendee | Problems encountered | Other note | | | |
|  |  | |  |  |  | | | |
| **Part II Call 4** | | | | - Completed □ Uncompleted | | | | |
| Date | Duration | | Attendee | Problems encountered | Other note | | | |
|  |  | |  |  |  | | | |
