# Supplementary material for: Effects of a family-focused dyadic psychoeducational intervention for stroke survivors and their family caregivers: a pilot study
Source: BMC Nurs. 2022 Dec 21;21:364. doi: 10.1186/s12912-022-01145-0 (PMC9768401; doi:10.1186/s12912-022-01145-0)
Supplement: Supplementary file 2 — Additional file 2: Appendix B. Intervention fidelity checklist. [file 12912_2022_1145_MOESM2_ESM.docx]

Appendix B Intervention fidelity checklist

| **Sessions and contents** | Completion status | |
| --- | --- | --- |
|  | Yes | No |
| **Part I Session 1** |  |  |
| 1. To provide overview of the intervention programme |  |  |
| 2. To provide basic information about stroke |  |  |
| 3. To clarify the misunderstanding about stroke-related knowledge for participants |  |  |
| **Part I Session 2** |  |  |
| 1. To provide information about post-stroke rehabilitation |  |  |
| 2. To provide information about care for survivors |  |  |
| 3. To provide information about care for caregivers |  |  |
| 4. To facilitate rehabilitation and care/selfcare technique training |  |  |
| 5. To discuss care-related issues encountered by participants and related coping strategies |  |  |
| **Part I Session 3** |  |  |
| 1. To present post-stroke stress and psychological disturbances |  |  |
| 2. To provide information on how to cope with post-stroke stress and psychosocial disturbances |  |  |
| 3. To facilitate the technique training of stress management and relaxation |  |  |
| 4. To discuss participants’ post-stroke psychosocial experience and coping strategies |  |  |
| **Part II Call 1** |  |  |
| To discuss at least one problems or issues encountered in their daily life |  |  |
| **Part II Call 2** |  |  |
| To discuss at least one problems or issues encountered in their daily life |  |  |
| **Part II Call 3** |  |  |
| To discuss at least one problems or issues encountered in their daily life |  |  |
| **Part II Call 4** |  |  |
| To discuss at least one problems or issues encountered in their daily life |  |  |
