# Supplementary material for: Effects of a family-focused dyadic psychoeducational intervention for stroke survivors and their family caregivers: a pilot study
Source: BMC Nurs. 2022 Dec 21;21:364. doi: 10.1186/s12912-022-01145-0 (PMC9768401; doi:10.1186/s12912-022-01145-0)
Supplement: Supplementary file 3 — Additional file 3: Appendix C. The open-ended questions of qualitative interview. [file 12912_2022_1145_MOESM3_ESM.docx]

Appendix C. The open-ended questions of qualitative interview

| Questions |
| --- |
| 1. What do you think and feel about the experience of the intervention? |
| 1. Describe any experience that are very impressive to you. 2. Any part you feel very positive or negative? Any reasons suggested? |
| 1. What aspects of this intervention do you think to be beneficial/useful to you? 2. Which one is most useful? Please explain. 3. What do you feel about the structure and format of the sessions? Which parts are found appropriate and well-structured? |
| 1. What aspects of this intervention do you think are unhelpful or should be improved? 2. Which is not or least helpful? Please explain. 3. What parts of the structure and sessions do you think are inappropriate? |
| 1. What aspects of this intervention do you think are difficult for you to understand or adopt? |
| 1. Which parts do you think are difficult for you to understand? Please describe the details. 2. Which parts do you think are difficult for you to adopt? Any reason suggested? |
| 1. Any other suggestions for the interventions? |
